# Weight-Bearing and Mobilization in the Postoperative Care of Ankle Fractures: A Systematic Review and Meta-Analysis of Randomized Controlled Trials and Cohort Studies

**DOI:** 10.1371/journal.pone.0118320

**Published:** 2015-02-19

**Authors:** Diederik P. J. Smeeing, Roderick M. Houwert, Jan Paul Briet, Johannes C. Kelder, Michiel J. M. Segers, Egbert Jan M. M. Verleisdonk, Luke P. H. Leenen, Falco Hietbrink

**Affiliations:** 1 Department of Surgery, University Medical Center Utrecht, Utrecht, The Netherlands; 2 Traumacenter University Medical Center Utrecht, Utrecht, The Netherlands; 3 Department of Surgery, Diakonessenhuis Utrecht, Utrecht, The Netherlands; 4 Department of Epidemiology, St Antonius Hospital Nieuwegein, Nieuwegein, The Netherlands; 5 Department of Surgery, St Antonius Hospital Nieuwegein, Nieuwegein, The Netherlands; University of Toledo, UNITED STATES

## Abstract

**Purpose:**

To determine the effectiveness and safety of interventions used for rehabilitation after open reduction and internal fixation of ankle fractures.

**Methods:**

A systematic review and meta-analysis was performed using both randomized trials and cohort studies. The effect of mobilization, weight-bearing, and unprotected weight-bearing as tolerated on postoperative recovery was compared using the Olerud Molander score, return to work/daily activities, and the rate of complications.

**Results:**

A total of 25 articles were included. Ankle exercises resulted in earlier return to work and/or daily activities compared to immobilization (mean difference (MD) -20.76 days; 95% confidence interval (CI) -40.02 to -1.50). There was no difference in the rate of complications between exercises and immobilization (risk ratio (RR) 1.22; 95% CI 0.60 to 2.45) or between early and late weight-bearing (RR 1.26; 95%CI 0.56 to 2.85).

**Interpretation:**

Results of this meta-analysis show that following ankle surgery, 1) active exercises accelerate return to work and daily activities compared to immobilization, 2) early weight-bearing tends to accelerate return to work and daily activities compared to late weight-bearing. Active exercises in combination with immediate weight-bearing may be a safe option.

## Introduction

Ankle fractures are the most common type of lower extremity fracture and among the most common types of fractures worldwide [1]. The incidence of ankle fractures is between 100 and 150 per 100,000 person-years and rising [2,3]. More than half of ankle fractures occur during sports activities, which is indicative of a healthy patient population [4].

Ankle fractures are associated with high costs for both the affected individual and society. These costs are related not only to the operation and subsequent hospitalization, but also to the duration of occupational disability [5–8]. To reduce these latter costs, early functional return is crucial [9].

While various classification systems exist to describe ankle fractures, the indication for surgery depends on the congruency of the ankle joint [10–12]. When an incongruent joint is present, fractures are often treated by open reduction and internal fixation to stabilize the ankle joint. The postoperative care regimens vary widely, e.g. from immobilization in a plaster cast without weight-bearing for multiple weeks to immediate post-operative protected mobilization. Even direct post-operative unprotected weight-bearing as tolerated has been suggested [13].

Several systematic reviews have been published on various aspects of postoperative care regimens [14–17]. However, none of these reviews could provide clear advice for the postoperative care of ankle fractures since the regimens differ in terms of protection, ankle movements, and weight-bearing. In this systematic review and meta-analysis, non-randomized studies were included to potentially assist in showing effectiveness. Furthermore, specific aspects of the post-operative care regimen were compared.

The aim of this study was to determine the effectiveness and safety of interventions that are used for rehabilitation after open reduction and internal fixation of ankle fractures.

## Methods

A published review protocol does not exist.

### Search strategy and selection criteria

Randomized controlled trials (RCT’s) and cohort studies evaluating postoperative care regimen of ankle fractures were included in this systematic review and meta-analysis (Weber, AO or Lauge Hansen classifications) [10,11]. Two reviewers (DS and FH) independently searched the MEDLINE and EMBASE electronic databases in January 2014. No language or publication restrictions were applied to the search, and disagreements were resolved by discussion (DS, FH and RH). The search was conducted using the following specific broad search string: [ankle fracture OR Lauge Hansen OR Weber] AND [cast OR orthosis OR weight-bearing] AND [Olerud Molander OR follow-up].

### Quality assessment

The methodological quality of all included studies was assessed by three reviewers (DS, FH and RH) using the ‘risk of bias’ assessment tool provided by The Cochrane Collaboration [18].

### Outcome measures

The effectiveness and safety of the postoperative care regimens were evaluated using the following outcome measures: the Olerud Molander score for functional objective outcome (short-term and long-term) [19], return to work and/or daily activities in days for functional subjective outcome, and rate of complications, which is a direct result of the postoperative care regimen, for safety outcome.

### Data extraction

Two reviewers (DS and FH) independently extracted the following data: authors, year of publication, study design, treatment groups, outcome, results between 6 and 12 weeks (short-term), results after 6 months (long-term), confidence interval (CI) or *P* value, and complications. The complications were defined as reported in the studies. Author contact was attempted for studies with missing data.

### Data analysis

Studies were included in the short-term analysis for the Olerud Molander score if results between 6 and 12 weeks were reported. If more short-term results were reported, the results closest to 6 weeks were used. Studies were included in the long-term analysis if results after 6 months were reported. If more results were reported on the long-term, the Olerud Molander scores from the final survey were used.

Review Manager ((RevMan) [Computer program]. Version 5.2. Copenhagen: The Nordic Cochrane Centre, The Cochrane Collaboration, 2012.) was used for the meta-analysis. A mean difference (MD) was calculated for continuous outcomes. The Inverse Variance (IV) with fixed effect (Fixed) or with random effect (Random) method was used to construct a 95%CI. If a study did not report standard deviation (SD), the value was not estimated. Available case analysis was performed with missing data. A risk ratio (RR) was calculated for complications, which was the only dichotomous outcome. The Mantel-Haenszel (M-H) with fixed effect (Fixed) or random effect (Random) method was used to compute a 95%CI for the dichotomous outcome. The assessment of statistical heterogeneity was done by visual inspection of the forest plots, the Tau^2^ test, the Chi^2^ test, the degrees of freedom (df), the I^2^ value and the overall effect Z-test. If the I^2^ value was below 25%, fixed effects were used. If the I^2^ value was 25% or greater, random effects were used. After the primary analyses, sensitivity analyses were performed. In the sensitivity analyses on bias, all studies with less than 5 high risk factors for bias were included. Studies with 5 or more high risk factors for bias were excluded. In the sensitivity analysis on study design, only RCT’s were included. In the sensitivity analyses on time, all studies published after 2000 were included.

## Results

### Search

The MEDLINE and EMBASE search identified 259 and 87 studies, respectively. The 14 relevant studies from the initial search were checked for related citations, resulting in 28 relevant studies.

**Fig 1:**
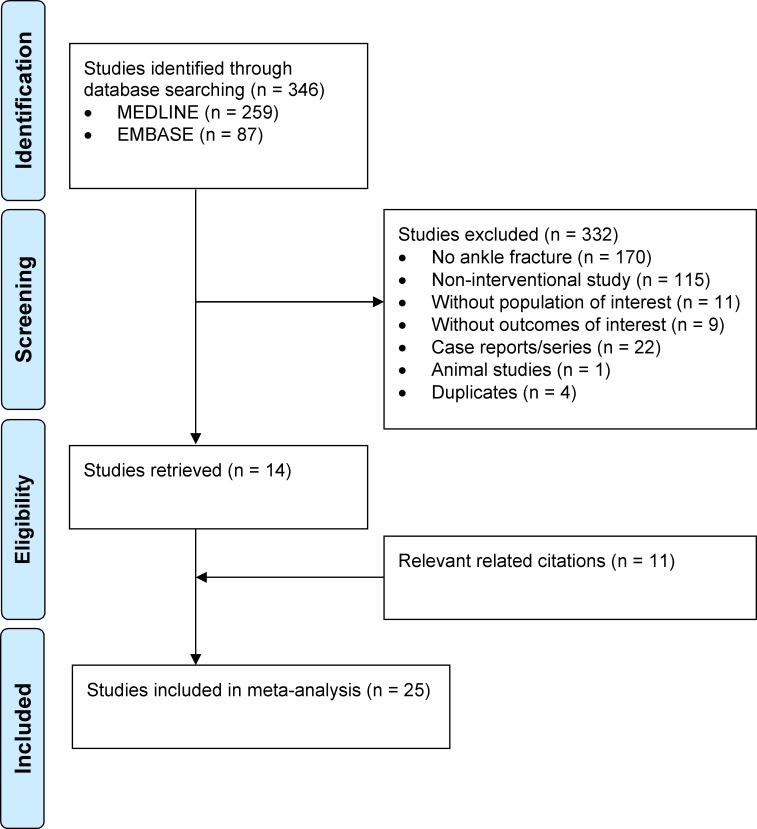
Process from initial search to final inclusion of studies on postoperative care of ankle fractures. A flowchart following the PRISMA guidelines of the search strategy.

In Kimmel et al. mobilization started with a difference of one day between the two groups and in Starkweather et al. with a difference of seven days [20,21]. Partenheimer et al. only analysed one group of patients [22]. Therefore, these studies were not included. Ultimately, a total of 25 studies met the inclusion criteria (Fig. 1) [13,23–46].

### Baseline characteristics

Of the 25 included studies, 12 were RCT’s, 7 were prospective studies, 1 was a retrospective study, and 5 were partly prospective and partly retrospective studies. A total of 1376 participants were included. Studies included in this meta-analysis did not describe the classification of ankle fractures (AO, Weber or Lauge Hansen), age, or medical co-morbidities in relation to the different outcomes (S1 Table).

### Bias

**Fig 2:**
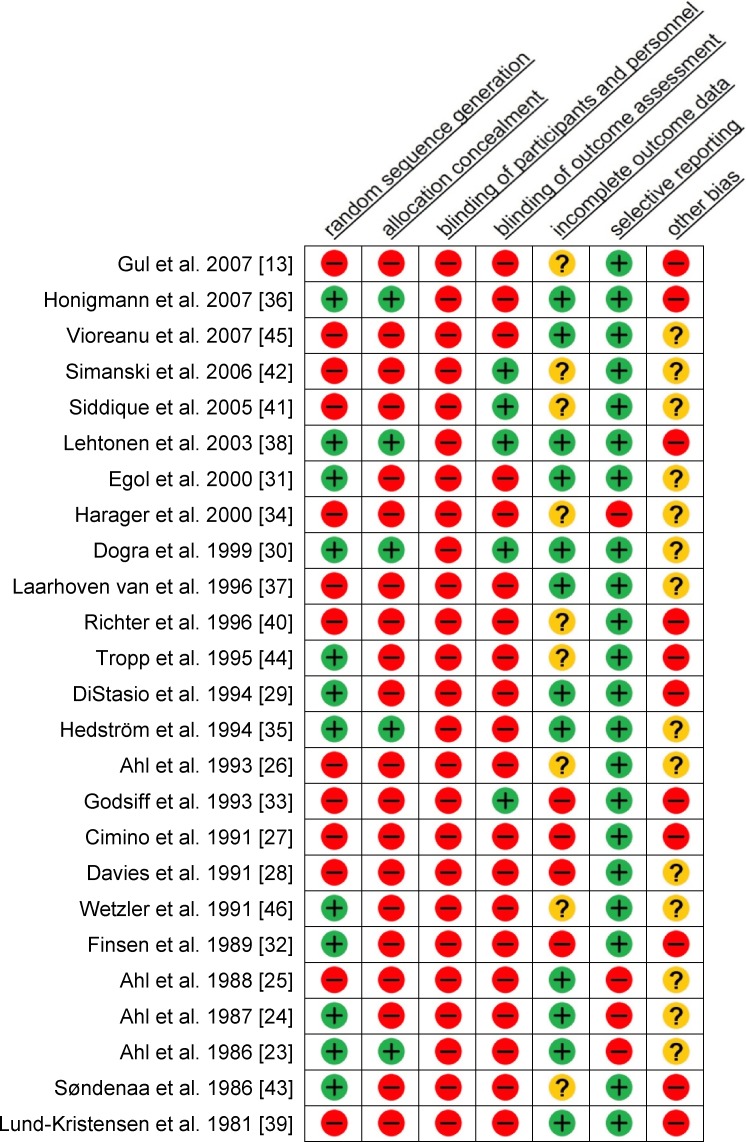
Risk of bias assessment. —indicates a high risk of bias; + indicates a low risk of bias;? indicates an unclear risk of bias.

Nearly all studies had a high risk of bias, both cohort studies and RCT’s (Fig. 2). More than half of the studies had a high risk on random sequence generation (52%), on allocation concealment (80%), and on blinding of outcome assessment (80%). Since treatment was visible, the participants and personnel of all studies, cohort studies and RCT’s, were not blinded. In 11 (44%) studies, a high risk of other bias was found. In 7 of these studies, patients with a Weber A fracture who had undergone surgery were included [13,27,33,36,38,39,43]. However, further specifications of these fractures were not presented.

### Effect of mobilization

A total of 19 studies investigated the effect of mobilization (ankle exercises) on recovery [13,27–33,35–38,40–46]. Honigmann et al. and Laarhoven van et al. only reported the median results and Wetzler et al. did not describe enough data for analysis [36,37,46]. Therefore, these three studies were excluded from the analysis. Tropp et al. used a modified Olerud Molander score [44]. Finsen et al. compared 3 groups with each other; only groups A and B with a non-weight-bearing treatment were analysed [32].

The Olerud Molander score was reported in 9 studies. No statistically significant results were found with the short-term (MD -6.88; 95%CI -17.66 to 3.91) or long-term Olerud Molander score (MD -1.05; 95%CI -4.11 to 2.00).

**Fig 3:**
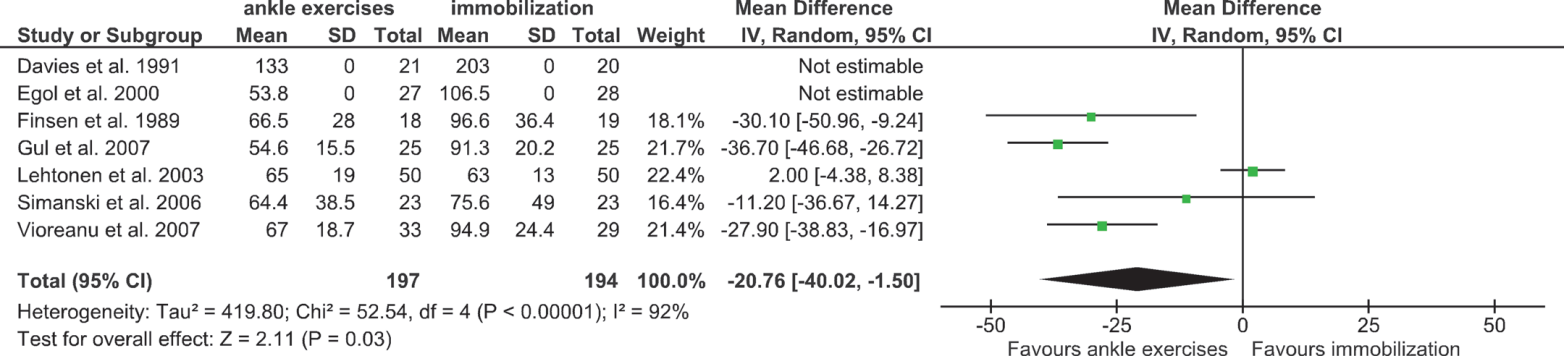
Effect of mobilization; return to work/daily activities. The mean difference (SD) on the return to work/daily activities is shown between the ankle exercises groups and the immobilization groups.

The return to work and/or daily activities was reported in 7 of the active mobilization studies (Fig. 3). The total mean difference was -20.76 days in favour of the ankle exercises group (95%CI -40.02 to -1.50).

Complications were reported entirely (and attributed to a group) in 12 studies. The nature and definition of the complications differed, but they were comparable in terms of severity. No statistically significant difference was found in the occurrence of complications (RR 1.22; 95%CI 0.60 to 2.45). Lehtonen’s data skewed the results [38].

Unprotected mobilization was described in 7 studies [13,32,36,37,39,41,43]. With the exception of Gul et al., these studies used unprotected mobilization in combination with late weight-bearing (3–6 weeks post-operatively) [13]. Finsen et al. and Lund-Kristensen et al. did not report the groups in which complications occurred [32,39]. Siddique et al. did not report the complications [41]. No statistically significant difference was found in the occurrence of complications (RR 1.03; 95%CI 0.42 to 2.50).

### Effect of weight-bearing

The effect of weight-bearing on postoperative recovery was reported in 11 studies that compared early versus late weight-bearing [13,23–26,32,34,36,37,42,46]. Honigmann et al. and Laarhoven van et al. reported only the median scores, and Wetzler et al. did not describe enough data for analysis. Therefore, these three studies were excluded from analyses [36,37,46].

Analysis of the short-term Olerud Molander score was not possible. Although the long-term Olerud Molander score was reported in three studies, only Simanski et al. reported the SD values, so analysis of the long-term score also was not possible [42].

**Fig 4:**
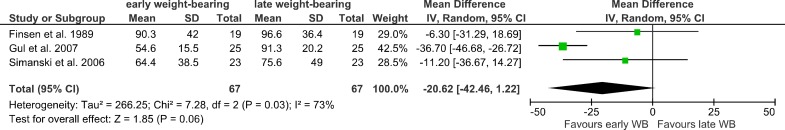
Effect of weight-bearing; return to work/daily activities. The mean difference (SD) on the return to work/daily activites is shown between the early weight-bearing groups and the late weight-bearing groups.

Of the 11 studies, only 3 reported the mean results of return to work and/or daily activities (Fig. 4). In Finsen et al., the two groups without ankle exercises (groups B and C) were used in the analysis [32]. The total mean difference was -20.62 days in favour of the early weight-bearing group (95%CI -42.46 to 1.22). For analysis of complications, 8 of the 11 studies were used. No statistically significant difference was found (RR 1.26; 95%CI 0.56 to 2.85). Weight-bearing as tolerated began immediately after surgery in 3 studies [13,27,34]. Harager et al. only reported the complications of one group [34]. Therefore, only 2 of the 3 studies were analyzable. There was no statistically significant difference in the occurrence of complications (RR 0.69; 95%CI 0.28 to 1.73).

### Unprotected weight-bearing as tolerated immediately after surgery

Gul et al. was the only study to report unprotected immediate weight-bearing as tolerated [13]. No significant differences were found between the groups in the Olerud Molander score, postoperative pain intensity, and length of hospital stay. Analysis of return to work/daily activities resulted in a statistically significant mean difference of 36.7 days in favour of the unprotected immediate weight-bearing as tolerated group.

### Sensitivity analyses

In the sensitivity analyses on bias, 9 studies with 5 or more high risk factors for bias were excluded [13,25,27,28,32–34,39,40]. All analyses were repeated without these studies. The results remained in favour of the same group but lost their statistical significance, indicating insufficient power to demonstrate effectiveness if only high quality studies are used (Table 1). In the sensitivity analyses on study design about the effect of mobilization on the occurrence of complications, the result became significant (RR = 3.38; 95%CI = 1.82 to 6.28). The other analyses on study design showed no significant differences. In the sensitivity analyses on time, the results remained in favour of the same group and the effect of weight-bearing on the return to work/daily activities became significant (MD -26.75; 95%CI -51.13 to -2.37).

**Table 1:** Results of sensitivity analyses.

Outcome		Results	Sensitivity analysis on bias	Sensitivity analysis on study design	Sensitivity analysis on time
Effect of mobilization	Olerud Molander Score on short-term*	-6.88 [-17.66; 3.91]	-6.88 [-17.66; 3.91]	0.06 [-7.18; 7.31]	-6.84 [-19.87; 6.19]
	Olerud Molander Score on long-term*	-1.05 [-4.11; 2.00]	-1.05 [-4.11; 2.00]	-0.28 [-3.50; 2.94]	-2.67 [-10.06; 4.72]
	Return to work/daily activities in days*	**-20.76 [-40.02; -1.50]**	-12.18 [-34.89; 10.53]	-12.45 [-43.75; 18.85]	-18.68 [-40.70; 3.33]
	Complications**	1.22 [0.60; 2.45]	2.09 [0.93; 4.70]	**3.38 [1.82; 6.28]**	1.71 [0.67; 4.37]
	Complications; unprotected treatment**	1.03 [0.42; 2.50]	1.05 [0.30; 3.66]	3.43 [0.15; 79.74]	***
Effect of weight-bearing	Return to work/daily activities in days*	-20.62 [-42.46; 1.22]	***	***	**-26.75 [-51.13; -2.37]**
	Complications**	1.26 [0.56; 2.85]	1.41 [0.46; 4.36]	1.05 [0.06; 17.81]	1.00 [0.41; 2.45]
	Complications; weight-bearing as tolerated immediately after surgery**	0.69 [0.28; 1.73]	***	***	***

* means that results are presented as mean difference [95%CI].

** means that results are presented as risk ratio [95%CI].

*** means that ≤ one study can be included, therefore analysis cannot be performed.

## Discussion

Results of this systematic review and meta-analysis on a combination of cohort studies and randomized controlled trials show that after ankle surgery, 1) active exercises accelerate return to work and daily activities compared to immobilization, 2) early weight-bearing tends to accelerate return to work and daily activities compared to late weight-bearing. Furthermore, active exercises in combination with immediate weight-bearing may be a safe option that needs to be further explored.

These conclusions are based on 25 studies, 18 of which are quasi-randomized trials used in the recently published Cochrane meta-analysis of Lin et al. [47]. The Cochrane meta-analysis found limited evidence supporting early commencement of ankle exercises and weight-bearing, as it resulted in a reduced activity limitation score and an improved range of motion. However, there was also an increase in complication rates. A limitation of the Cochrane meta-analysis is that only randomized and quasi-randomized controlled trials were included, and as a result, most analyses were performed on 1–3 studies. This leads to a reduced possibility to show effectiveness. In contrast, this meta-analysis also included other study designs, which has been suggested by previous surgical and orthopaedic literature [48,49]. The randomization process in surgical trials is cumbersome and creates an artificial situation that may deviate from “normal” clinical practice substantially [50]. Therefore, results of this meta-analysis are more likely to correspond to “standard” clinical practice, despite all the drawbacks of non-randomized clinical research. This concept has been initiated in the field of surgery and we extrapolated this in the postoperative treatment. The patients cannot be blinded for their treatment regime and return to work or daily activities is a patient subjective outcome measure. Sensitivity analyses, which do not show a change in direction of the results after correcting for study quality, support this phenomenon. The sensitivity analysis according to study design for complications changed into statistical significance in favour of immobilization. These results are heavily influenced by the study of Lehtonen et al. [38].

This study provides data about return to work, which is becoming increasingly important for health care providers in current practice. We found that active mobilization results in earlier return to work and/or daily activities of 21 days. This result might lead to a cost reduction in social security payments and lost wages [5,7,51].

Other reviews have been published on various aspects of postoperative care regimens [14–17]. Nash et al. concluded that immobilization provides no benefit, and Thomas et al. concluded that it is difficult to determine whether early motion is overall better or worse than cast immobilization [15,17]. Black et al. suggested that early weight-bearing might allow for faster rehabilitation and earlier return to work [14]. Smith et al. concluded that because of the little difference in outcome, the optimum time to commence weight-bearing cannot be determined [16]. These reviews focused on whole postoperative care regimens rather than specific aspects of postoperative care and could not give a differentiated clinical advice. In this review, we analyzed separate aspects of the postoperative care regimen: effect of ankle exercises, weight-bearing, and unprotected treatment in order to tailor clinical advice accordingly.

Nilsson et al. suggested that a difference of 4.4 points in the Olerud Molander score is of clinical interest for a group of patients [52]. This meta-analysis shows an even greater difference in the Olerud Molander score of 6.88 points on short-term in favour of active mobilization and 8.00 points on long-term in favour of early weight-bearing. Both outcomes fail to reach statistical significance but are in concordance with previous analyses [15,17,47].

Due to the inclusion of all relevant study designs without language restrictions, a large number of studies were evaluated in this meta-analysis. Three independent reviewers systematically evaluated the studies for risk of bias. In this quality assessment, a great deal of bias was found in the published studies, which rendered the results of these studies less reliable. The different variables used in this meta-analysis could cause heterogeneity because of poor definitions of these variables in some of the included studies. This meta-analysis describes the complications as defined in the studies, which allowed the severity of complications to be compared. However, definitions vary and thus may result in bias.

Direct active mobilization after surgery is advantageous compared to immobilization, and weight-bearing shows a similar trend. This meta-analysis shows no increased risk for complications with active mobilization after early weight-bearing. Therefore, the combination of active mobilization and early weight-bearing might be a promising strategy. Gul et al. published encouraging results on direct unprotected weight-bearing as tolerated in a retrospective study design [13]. Currently, there are two trials being conducted on the postoperative care of ankle fractures (NCT01196338 and NTR3727).

## Conclusion

Active mobilization results in an earlier return to work. Both active mobilization and immediate protected weight-bearing may be safe postoperative care strategies after ankle surgery, so the regimen choice should be based on patient demands with an emphasis on active mobilization. Unprotected weight-bearing as tolerated after ankle surgery may also be a safe and promising choice. As such, future research should address this option.

## Supporting Information

S1 Table Characteristics of included studies. Results which are not used in analyses are written in italics. Complications that are not directly related to the post-operative treatment regimen and the complications with an unknown group are also written in italics. + means yes/present; - means no/not present.
(PDF)

S2 Table PRISMA Checklist. (PDF)
