# Supplementary material for: Weight-Bearing and Mobilization in the Postoperative Care of Ankle Fractures: A Systematic Review and Meta-Analysis of Randomized Controlled Trials and Cohort Studies
Source: PLoS One. 2015 Feb 19;10(2):e0118320. doi: 10.1371/journal.pone.0118320 (PMC4335061; doi:10.1371/journal.pone.0118320)
Supplement: S1 Table — Results which are not used in analyses are written in italics. Complications that are not directly related to the post-operative treatment regimen and the complications with an unknown group are also written in italics. + means yes/present; - means no/not present. (PDF) [file pone.0118320.s001.pdf]

S1 Table Characteristics of included studies

Publication and study design	Groups (n = number of patients)	Weight-bearing	Mobili-zation	Protection			Outcome	Results 6-12 weeks	Results ≥ 6 months	*P* value	Complications	Details bias assessment
				Cast	Orthosis	Unpro-tected						
**Ahl et al. 1986 [23] Randomized controlled trial**	(A) Late weight-bearing (starting from the fourth postoperative week) in a below-knee cast (n = 22)<br>vs<br>(B) Early weight-bearing (starting from the first post-operative day) in a below-knee cast (n = 24)	-<br><br>+	-<br><br>-	+<br><br>+	-<br><br>-	-<br><br>-	Olerud Molander score	Not reported	Not reported	Not reported	Group A: 2 older patients with osteoporosis revealed a redislocation of the lateral malleolus on radiograms	The Olerud Molander score is mentioned in the methods but the results are not reported (high risk on selective reporting).
**Ahl et al. 1987 [24] Randomized controlled trial**	(A) Late weight-bearing (starting from the fourth postoperative week) in a below-knee cast (n = 28)<br>vs<br>(B) Immediate weight-bearing (starting from the first post-operative day) in a below-knee cast (n = 25)	-<br><br>+	-<br><br>-	+<br><br>+	-<br><br>-	-<br><br>-	Olerud Molander score	Not reported	Not reported	Not reported	Group A (2) and Group B (6) cases of superficial wound infection or skin irritation<br>The difference between the 8 cases was not significant<br><br>*Group B: 1 case redislocation (caused by a new trauma)*	The Olerud Molander score is mentioned in the methods but the results are not reported (high risk on selective reporting).
**Ahl et al. 1988 [25] Randomized controlled trial and retrospective comparisons to Ahl et al. 1986**	(A) Ankle exercises without weight-bearing in a dorsal splint (n=25)<br>vs<br>(B) Ankle exercises with weight-bearing in an orthosis (n=26)<br>Both groups were initially treated with a plaster cast without weight-bearing during the first postoperative week	-<br><br>+	+<br><br>+	+<br><br>-	-<br><br>+	-<br><br>-	Olerud Molander score	Not reported	Not reported	Not reported	No complications	The Olerud Molander score is mentioned in the methods but the results are not reported (high risk on selective reporting).
**Ahl et al. 1993 [26] Randomized controlled trial and retrospective comparisons to Ahl et al. 1987**	(A) Ankle exercises without weight-bearing in a dorsal splint (n=19)<br>vs<br>(B) Ankle exercises with weight-bearing in an orthosis (n=21)<br>Both groups were initially treated with a plaster cast without weight-bearing during the first postoperative week	-<br><br>+	+<br><br>+	+<br><br>-	-<br><br>+	-<br><br>-	Olerud Molander score	Group A = 53<br>Group B = 66<br>(at 3 months)	*Group A = 76*<br>*Group B = 82*<br>*(at 6 months)*<br><br>Group A = 86<br>Group B = 90<br>(at 18 months)	Not reported<br><br>Not reported<br><br>Not reported	Group B: 3 cases with superficial wound infection, 1 case with local wound necrosis and 1 case with skin irritation, caused by the orthosis	

Publication and study design	Groups (n = number of patients)	Weight-bearing	Mobili-zation	Protection			Outcome	Results 6-12 weeks	Results ≥ 6 months	*P* value	Complications	Details bias assessment
				Cast	Orthosis	Unpro-tected						
**Cimino et al. 1991 [27] Partly prospective study (63/71), partly retrospective study (8/71)**	(A) Immobilization in a cast (immediate full weight-bearing)<br>(n=19)<br>vs<br>(B) Ankle-foot orthosis (immediate full weight-bearing) with ankle exercises<br>(n=32)	+<br>+	-<br>+	+<br>-	-<br>+	-<br>-	---	---	---	---	Group A: 2 cases with wound complication<br>1 case with painful bursa (reoperation)<br>1 case with hardware loosening (reoperation)<br>Group B: 2 cases with wound complication<br>1 case with widening of the malleoli due to syndesmosis injury<br><br>*Group B: 1 case with a femoral-neck fracture and a pulmonary embolism*	Eight patients are previously reported (high risk on other bias).<br>Weber A fractures are included (high risk on other bias).<br>Three patients are mistakenly assigned to group B (high risk on other bias).
**Davies et al. 1991 [28] Prospective study**	(A) Immobilization in a below-knee plaster of Paris<br>(n = 20)<br>vs<br>(B) Ankle exercises and continuous passive movements (after 24 hours in a below-knee plaster of Paris backslab)<br>(n = 21)<br>Both groups were non-weight-bearing	-<br>-	-<br>+	+<br>-	-<br>-	-<br>+	Return to work (in days)	Group A = 203<br>Group B = 133	---	p = 0.084	Group A:3 cases with a wound infection<br>Group B:2 cases with a wound infection<br><br>*Group A:2 cases with an early removal of metal*<br>*1 case with osteoporosis*<br>*Group B: 6 cases with an early removal of metal*<br>*2 cases with osteoporosis*<br>*1 case with a combination of above described complications*	
**DiStasio et al. 1994 [29] Randomized controlled trial**	(A) Immobilization in a below-knee short-leg cast<br>(n = not reported)<br>vs<br>(B) Physical therapy immediately after surgery in a removable short-leg orthosis<br>(n = not reported)<br>Both groups were non-weight-bearing for 6 weeks<br>(total n = 61)	-<br>-	-<br>+	+<br>-	-<br>+	-<br>-	Return to work (in days)	Group A = 186<br>Group B = 174	---	Reported as not significant	Group A:2 cases with a superficial wound infection<br>Group B:1 case with a superficial wound infection<br><br>*Group unknown:5 cases with a removal of internal fixation hardware for local symptomatology*	The results are reported for only 34% of the patients (high risk on other bias).<br>The total number of patients is reported, but not how many of these patients are assigned to the different treatment groups (high risk on other bias).

Publication and study design	Groups (n = number of patients)	Weight-bearing	Mobili-zation	Protection			Outcome	Results 6-12 weeks	Results ≥ 6 months	*P* value	Complications	Details bias assessment
				Cast	Orthosis	Unpro-tected						
**Dogra et al. 1999 [30] Randomized controlled trial**	(A) Non-weight-bearing in a plaster cast in the first two weeks postoperatively without ankle exercises<br>(n=26)<br>vs	-	-	+	-	-	Olerud Molander score	Group A = 43.4 (30 - 65)<br>Group B = 46.2 (35 - 60)<br>(at 12 weeks)	---	Reported as not significant	*Group unknown: 1 case of superficial wound infection*	
	(B) Non-weight-bearing with ankle exercises in the first two weeks postoperatively. The ankle was resting in a plaster slab at all other times.<br>(n=26)<br>In both groups a below-knee plaster cast was applied after two weeks, graduated weight-bearing was instructed.	-	+	+	-	-						
**Egol et al. 2000 [31] Randomized controlled trial**	(A) Non-weight-bearing fibre-glass short-leg cast without ankle exercises<br>After 6 weeks patients started physiotherapy<br>(n=28)<br>vs	-	-	+	-	-	Return to work (in days)<br>(35 out of 55 patients)	Group A = 106.5<br>Group B = 53.8	---	p = 0.007	Group A: 1 case with pulmonary embolus	
	(B) Non-weight-bearing in a removable functional brace (aircast) with active and passive exercises of the ankle and subtalar joint by a physiotherapist<br>Patients continued the ankle exercises at home<br>(n=27)<br>Both groups were initially treated for the first 2 or 3 days with a plaster A-O splint<br>Both groups started weight-bearing at 6 weeks	-	+	-	+	-						
**Finsen et al. 1989 [32] Randomized controlled trial**	(A) Non-weight-bearing in a light-weight plaster-of-Paris cast<br>(n=19)<br>vs	-	-	+	-	-	Return to work (in days)	Group A = 96.6 (SD = 36.4)<br>Group B = 66.5 (SD = 28)<br>Group C = 90.3 (SD = 42)	---	reported as not significant	Group C: 1 case with readmission because of swelling, irritation of the skin and blistering<br><br>*Group unknown:2 cases with a superficial wound infection*<br>*1 case with a deep wound infection*<br>*3 cases with a superficial infection or a small area of necrosis of the skin after removal of the plate and screw.*	The total loss to follow-up is reported, but not how the loss to follow-up is divided between the different treatment groups (high risk on other bias).
	(B) Non-weight-bearing without a plaster cast (first three days in a plaster-of-Paris splint) with ankle exercises<br>(n=18)<br>vs	-	+	-	-	+						
	(C) Weight-bearing in a below-knee cast with a rubber walker<br>(n=19)	+	-	+	-	-						

Publication and study design	Groups (n = number of patients)	Weight-bearing	Mobili-zation	Protection			Outcome	Results 6-12 weeks	Results ≥ 6 months	*P* value	Complications	Details bias assessment
				Cast	Orthosis	Unpro-tected						
**Godsiff et al. 1993 [33] Prospective study**	(A) No early ankle exercises in a non-weight-bearing cast for 6 weeks<br>(n = 20)<br>vs	-	-	+	-	-	---	---	---	---	Not reported	Weber A fractures are included (high risk on other bias).
	(B) Early active and passive ankle exercises in a non-weight-bearing cast for 6 weeks<br>(n = 27)	-	+	+	-	-						
**Gul et al. 2007 [13] Retrospective study**	(A) Immobilization and non-weight-bearing in a plaster cast<br>(n=25)<br>vs	-	-	+	-	-	Olerud Molander score	---	Group A = 84<br>Group B = 82<br>(at an average 37,5 months)	p = 0.858	Group A: 1 case with superficial wound infection<br>1 case with a pulmonary embolism<br>1 case with loss of internal fixation at one week which was re-operated<br>1 case with non-union who refused re-operation<br>Group B: 3 cases with superficial wound infection who were put in an ankle brace to limit dorsal and plantar flexion for 1-2 weeks but were allowed to continue with full weight-bearing<br>1 case of posttraumatic arthritis<br><br>*Group B: 2 cases of syndesmotic screw breakage*	Weber A fractures are included (high risk on other bias).
	(B) Immediate functional and full weight-bearing mobilization without a plaster cast<br>(n=25)	+	+	-	-	+	Return to work (in days)	Group A = 91.3 (SD = 20.2)<br>Group B = 54.6 (SD = 15.5)	---	p < 0.001		
**Harager et al. 2000 [34] Partly prospective and partly retrospective study**	(A) Non-weight-bearing for 3 weeks followed by 3 weeks of weight-bearing<br>(n = 73)<br>vs	-	-	+	-	-	---	---	---	---	Group A: Not reported<br>Group B: 5 cases with a superficial wound infection<br>1 case with a slightly dislocated fracture<br>2 cases with a deep venous thrombosis	The hospital stay is mentioned in the methods but the quantitative results are not reported (high risk on selective reporting). The complications of group A are also not reported (high risk on selective reporting).
	(B) Immediate full weight-bearing in a below-knee walking cast<br>(n = 62)	+	-	+	-	-						

Publication and study design	Groups (n = number of patients)	Weight-bearing	Mobili-zation	Protection			Outcome	Results 6-12 weeks	Results ≥ 6 months	*P* value	Complications	Details bias assessment
				Cast	Orthosis	Unpro-tected						
**Hedström et al. 1994 [35] Randomized controlled trial**	(A) Weight-bearing in a walking cast without ankle exercises (n=25)<br>vs<br>(B) Weight-bearing in an orthosis with ankle exercises (n=28)	+<br>+	-<br>+	+<br>-	-<br>+	-<br>-	Olerud Molander score	Group A = 70<br>Group B = 80<br>(at 3 months)	*Group A = 85*<br>*Group B = 85*<br>*(at 6 months)*<br><br>Group A = 88<br>Group B = 100<br>(at 18 months)	Reported as not significant<br><br>Reported as not significant<br><br>Reported as not significant	Group A: 1 case with superficial wound infection<br>Group B: 2 cases with superficial wound infection<br><br>*Group B: 4 cases with arthrosis*	
**Honigmann et al. 2007 [36] Randomized controlled trial**	(A) Functional postoperative treatment without external stabilization (full weight-bearing after 6 weeks)<br>(a plaster of Paris splint was applied for 2-4 days)<br>(n=22)<br>vs<br>(B) Weight-bearing in a vacuum orthosis (vacoped)<br>(full weight-bearing is allowed after 14 days)<br>(n=23)<br>Both groups had 15kg weight-bearing starting between 2nd and 4th postoperative day.<br>Both groups had free ankle movements	-<br>+	+<br>+	-<br>-	-<br>+	+<br>-	*Olerud Molander score (median)*<br><br>*Return to work (in days) (median)*	*Group A = 42.5*<br>*Group B = 42*<br>*(at 6 weeks)*<br><br>*Group A = 72*<br>*Group B = 69*<br>*(at 10 weeks)*<br><br>*Group A = 53*<br>*Group B = 37*	---<br><br>---<br><br>---	p = 0.46<br><br>p = 0.55<br><br>p = 0.79	No complications	Weber A fractures are included (high risk on other bias).
**Laarhoven van et al. 1996 [37] Prospective study**	(A) Non-weight-bearing functional unprotected mobilization and crutches (n=40)<br>vs<br>(B) Early weight-bearing in a below-knee walking plaster (n=41)<br>Both groups were initially treated for two to five days with a plaster cast postoperatively	-<br>+	+<br>-	-<br>+	-<br>-	+<br>-	*Olerud Molander score (median)*<br><br>*Return to work (in days) (median)*	*Group A = 40*<br>*Group B = 45*<br>*(at 10 days)*<br><br>*Group A = 50*<br>*Group B = 65*<br>*(at 6 weeks)*<br><br>*Group A = 80*<br>*Group B = 85*<br>*(at 3 months)*<br><br>*Group A = 79*<br>*Group B = 78*	---<br><br>*Group A = 95*<br>*Group B = 95*<br>*(at 1 year)*<br><br>---	p = 0.47<br><br>p = 0.02<br><br>p = 0.84<br><br>p = 0.90<br><br>p = 0.54	Group A: 2 cases with a superficial wound infection<br>1 case with osteitis<br>Group B: 4 cases with a superficial wound infection<br><br>*Group unknown: 3 cases with secondary dislocation after operation*<br>*1 case with delayed union*<br>*2 cases with Sudeck's dystrophy*	

Publication and study design	Groups (n = number of patients)	Weight-bearing	Mobili-zation	Protection			Outcome	Results 6-12 weeks	Results ≥ 6 months	*P* value	Complications	Details bias assessment
				Cast	Orthosis	Unpro-tected						
**Lehtonen et al. 2003 [38] Randomized controlled trial**	(A) Below-knee plaster cast; after 2 weeks partial weight-bearing in a fibreglass short leg walking cast and full weight-bearing at 4 weeks (n=50)<br>vs	+	-	+	-	-	Olerud Molander score	Group A = 54 (SD = 13)<br>Group B = 52 (SD = 14)<br>(at 6 weeks)		Reported as not significant	Group A: 4 cases with a superficial wound infection<br>2 cases with a deep-vein thrombosis<br>1 case with chronic dysesthesia of the skin<br>Group B: 16 cases with a superficial wound infection<br>4 cases with a deep wound infection<br>3 cases with dehiscence of the wound<br>3 cases with chronic dysesthesia<br>1 case with local skin necrosis<br>1 case with chronic allodynia<br>1 case with loss of internal fixation<br>1 case with refracture of the lateral malleolus<br><br>*Group A: 1 case with chronic skin irritation*<br>*Group B: 2 cases with chronic skin irritation*<br>*1 case with postspinal headache*	Weber A fractures are included (high risk on other bias).
	(B) Functional Air-Stirrup ankle brace (Aircast); after 2 weeks partial weight-bearing and full weight-bearing at 4 weeks<br>(n=50)	+	+	-	+	-		*Group A = 75 (SD = 14)*<br>*Group B = 75 (SD = 13)*<br>*(at 12 weeks)*		Reported as not significant		
									Group A = 89 (SD = 8)<br>*Group B = 88 (SD = 9)*<br>*(at 52 weeks)*	Reported as not significant		
									Group A = 87 (SD = 8)<br>Group B = 87 (SD = 9)<br>(at 2 years)	Reported as not significant		
							Return to work (in days)	Group A = 63 (SD = 13)<br>Group B = 65 (SD = 19)	---	Reported as not significant		
**Lund-Kristensen et al. 1981 [39] Prospective study**	(A) Dorsal plaster-of-Paris splint for 2 to 10 days (average 3 days) with ankle exercises after removal of the dorsal splint<br>(n=10)<br>vs	-	-	+	-	-	---	---	---	---	*Group unknown: 1 case with a small abscess over a loose screw*<br>*1 case with pes equines*	Weber A fractures are included (high risk on other bias).<br>Very low number of patients (n=28) are included (high risk on other bias).
	(B) Plaster-of-Paris cast (for 4 to 6 weeks postoperative); as soon as possible the patients were mobilized using crutches<br>(n=3)<br>vs	-	+	+	-	-						
	(C) Without any external fixation with ankle exercises<br>(n=15)<br>All groups became weight-bearing on average at 9 weeks.	-	+	-	-	+						
**Richter et al. 1996 [40] Prospective study**	(A) Plaster immobilization (non-weight-bearing)<br>(n=19)<br>vs	-	-	+	-	-	Olerud Molander score	---	Group A = 85.0<br>Group B = 92.4<br>(on average at 17,3 months)	Not reported	No complications	Children are included (high risk on other bias).
	(B) Early mobilization<br>(n=42)	-	+	-	-	-						

Publication and study design	Groups (n = number of patients)	Weight-bearing	Mobili-zation	Protection			Outcome	Results 6-12 weeks	Results ≥ 6 months	*P* value	Complications	Details bias assessment
				Cast	Orthosis	Unpro-tected						
**Siddique et al. 2005 [41] Prospective study**	(A) Immobilization in a below-knee plaster cast (n=22)<br>vs<br>(B) Mobilization without a cast (n=22)<br>Both groups were treated with partial weight-bearing at 4 weeks and gradually progressed to full weight-bearing	-<br>-	-<br>+	+<br>-	-<br>-	-<br>+	Olerud Molander score	Group A = 33.0 (SD = 19.0)<br>Group B = 39.2 (SD = 19.5)<br>(at 6 weeks)<br>*Group A = 67.5 (SD = 21.9)*<br>*Group B = 69.5 (SD = 22.5)*<br>*(at 12 weeks)*	---<br>---	p = 0.29<br>p = 0.76	Not reported	
**Simanski et al. 2006 [42] Partly prospective and partly retrospective study**	(A) Immobilization (no functional treatment) in a below-knee plaster cast without weight-bearing (starting in a below-knee splint until ankle swelling was reduced)<br>(n=23)<br>vs<br>(B) Early functional (active and passive ankle exercises) treatment in a brace (aircast) with immediate partial weight-bearing; full weight-bearing after 3 weeks<br>(n=23)	-<br>+	-<br>+	+<br>-	-<br>+	-<br>-	Olerud Molander score<br>Return to work (in days)	---<br>Group A = 75.6 (SD = 49.0)<br>Group B = 64.4 (SD = 38.5)	Group A = 79 (SD = 19)<br>Group B = 87 (SD = 14)<br>(at least at 12 months)	p = 0.25<br>p = 0.63	Group A: 1 case with pseudarthrosis of the fibula<br>2 cases with superficial wound infection<br>1 case with reflex sympathetic dystrophy<br>(p = 0.66)<br>Group B: 1 case with reflex sympathetic dystrophy<br>1 case with an allergic reaction to the chrome-nickel plate	
**Søndenaa et al. 1986 [43] Randomized controlled trial**	(A) Non-weight-bearing immobilization in a plaster cast<br>(n=23)<br>vs<br>(B) Unprotected non-weight-bearing with active ankle exercises (after 3 days of pain immobilization in a plaster slab)<br>(n=20)<br>Both groups were allowed full weight-bearing after 6 weeks	-<br>-	-<br>+	+<br>-	-<br>-	-<br>+	---	---	---	---	Group B: 1 case with a superficial wound infection	Weber A fractures are included (high risk on other bias).
**Tropp et al. 1995 [44] Randomized controlled trial**	(A) Immobilization in a plaster cast<br>(n = 15)<br>vs<br>(B) Early mobilization in an ankle brace<br>(n = 15)<br>In both groups weight-bearing was allowed	+<br>+	-<br>+	+<br>-	-<br>+	-<br>-	Modified Olerud Molander score	Group A = 70 (SD = 21)<br>Group B = 77 (SD = 19)<br>(at 10 weeks)	Group A = 88 (SD = 22)<br>Group B = 92 (SD = 10)<br>(at 12 months)	Reported as not significant<br>Reported as not significant	Group A: no complications<br>Group B: 1 case with a wound with slight secretion	Very low number of patients (n=30) are included (high risk on other bias).<br>The total number of patients is reported, but not how many of these patients are assigned to the different treatment groups (high risk on other bias).

Publication and study design	Groups (n = number of patients)	Weight-bearing	Mobili-zation	Protection			Outcome	Results 6-12 weeks	Results ≥ 6 months	*P* value	Complications	Details bias assessment
				Cast	Orthosis	Unpro-tected						
**Vioreanu et al. 2007 [45] Prospective study**	(A) Immobilization in a below-knee nonremovable fibreglass cast (non-weight-bearing) (n=29) vs	-	-	+	-	-	Olerud Molander score	Group A = 63.75 (SD = 9.19) Group B = 79.92 (SD = 11.32) (at 9 weeks)	---	p < 0.05	Group A: 2 cases with deep vein thrombosis of popliteal vein Group B: 1 case with a superficial wound infection	
	(B) Early mobilization in a removable fibreglass cast with active and passive ankle exercises (non-weight-bearing) (n=33)	-	+	+	-	-		*Group A = 81.07 (SD = 9.56) Group B = 93.17 (SD = 8.76) (at 12 weeks)*	---	Reported as not significant	1 case with break down and subsequent infection of the wound which needed removal of hardware	
	Both groups were initially treated for 10 to 14 days in a dorsal plaster of Paris splint						Return to work (in days)	Group A = 94.9 (SD = 24.4) Group B = 67 (SD = 18.7)	---	p < 0.05	1 case with a deep wound infection which needed removal of hardware	
**Wetzler et al. 1991 [46] *(abstract only)* Randomized controlled trial**	(A) Short leg cast for 6 weeks (n = 25) vs	-	-	+	-	-	Return to work (in days)	No difference between both groups	---	Reported as not significant	Number of complications in both groups are not significantly different	
	(B) Pneumatic walker for 1 to 2 weeks and then a pneumatic ankle brace (n = 20)	+	+	-	+	-						

Results which are not used in analyses are written in italics. Complications that are not directly related to the post-operative treatment regimen and the complications with an unknown group are also written in italics.
+ = yes / present
- = no / not present
